# Evaluating Neighborhood Correlates and Geospatial Distribution of Breast, Cervical, and Colorectal Cancer Incidence

**DOI:** 10.3389/fonc.2018.00471

**Published:** 2018-10-30

**Authors:** Aracelis Z. Torres, Darcy Phelan-Emrick, Carlos Castillo-Salgado

**Affiliations:** ^1^Department of Epidemiology, Bloomberg School of Public Health, Johns Hopkins, Baltimore, MD, United States; ^2^Baltimore City Health Department, Baltimore, MD, United States

**Keywords:** cancer disparities, geospatial analysis, social determinants of health, spatial clusters, cancer incidence

## Abstract

**Introduction:** Though cancer research has traditionally centered on individual-level exposures, there is growing interest in the geography of both cancer and its risk factors. This geographic and epidemiological research has consistently shown that cancer outcomes and their known causal exposures exhibit geographic variation that coincide with area-level socioeconomic status and the composition of neighborhoods. A retrospective study was conducted to evaluate geospatial variation for female breast, cervical, and colorectal cancer incidence in Baltimore City.

**Materials and Methods:** Using a Maryland Cancer Registry dataset of incident breast, cervical, and colorectal cancers (*N* = 4,966) among Baltimore City female residents diagnosed from 2000 to 2010, spatial and epidemiological analyses were conducted through choropleth maps, spatial cluster identification, and local Moran's I. Ordinary least squares regression models identified characteristics associated with the geospatial clusters.

**Results:** Each cancer type exhibited geographic variation across Baltimore City with the neighborhoods showing high incidence differing by cancer type. Specifically, breast cancer had significant low incidence in downtown Baltimore while cervical cancer had high incidence. The neighborhood covariates associated with the geographic variation also differed by cancer type while local Moran's I identified discordant clusters.

**Discussion:** Cancer incidence varied geographically by cancer type within a single city (county). Small area estimates are needed to detect local patterns of disease when developing health and preventative programs. Given the observed variability of community-level characteristics associated with each cancer type incidence, local information is essential for developing place-, social-, and outcome-specific interventions.

## Introduction

Population-level improvements in cancer-related outcomes have not been equitably distributed. For instance, although African-American females have a lower overall cancer incidence rate compared to white females, their rate has remained relatively unchanged from 2000 to 2009 even though white females experienced a 0.3% annual decline (1). From 2005 to 2009, African-American

females had a 34% higher occurrence of cervical cancer compared to white females (2, 3). Notably, this 34% difference was a conservative estimate since these figures did not correct for hysterectomy status, which is more prevalent among African-American females (4). Differences are also observed in local geospatial comparisons. When evaluating cancer incidence within the time period of 2009–2013, Baltimore City females had a significantly higher age-adjusted incidence of all cancer types (446.8 cases per 100,000 population per year) vs. the Maryland state rate (416.3 cases per 100,000 population per year) (2). Baltimore City was also higher for cervical and colorectal cancer among females with breast cancer having comparable rates.

Though cancer research has traditionally centered on the role of individual-level exposures, there is growing interest in the geography of both cancer and its risk factors (5–7). This geographic and epidemiological research has consistently shown that cancer outcomes and their known causal exposures exhibit geographic variation that coincide with area-level socioeconomic status and the composition of neighborhoods (8–12). This expansion of the disease paradigm within cancer has resulted in directing more attention toward not only the geographic distribution of biological factors but also of the social determinants of health (13–15).

The broadening of the medical model is relevant in two particular ways for cancer research. Firstly, it brings new perspective to the already known epidemiological exposure-outcome associations that have been studied at the individual-level. There is a growing understanding that these well-known associations may vary within different community settings. Secondly, it leads to a better grasp of how distal factors play a role on health outcomes and subsequently provides more upstream avenues potentially on which to intervene. This paper aims to evaluate the geographic distributions of breast, cervical, and colorectal cancer incidence among female residents in Baltimore City, Maryland as well as the neighborhood characteristics associated with those distributions.

## Materials and methods

### Cancer incidence ascertainment

The Maryland Cancer Registry (MCR) served as the data source for individual-level cancer incidence records. For inclusion, cancer cases needed to be a female resident of Baltimore City when diagnosed between the ages of 21 and 74 years from 2000 to 2010. Only cases that were classified as having breast, cervical, or colorectal cancer were included. Given the spatial nature of the analysis, cases were also required to have a street address that could be geocoded (i.e., assigned latitude and longitude coordinates). If the same individual developed cancer multiple times in the same primary tumor site (e.g., breast), only the first diagnosis was counted. For instances where the same individual had a primary diagnosis in multiple sites of interest (e.g., breast and colorectal), each tumor was counted separately and evaluated in the corresponding cancer type-specific model.

### Neighborhood characteristics

The 2012 Baltimore Neighborhood Indicators Alliance Vital Signs report was utilized for the independent neighborhood-level data that described the quality of life within each Community Statistical Area (CSA) (16). CSAs are groupings of census tracts that approximate Baltimore City neighborhoods. The Vital Signs public dataset characterized each of Baltimore City's 55 CSAs through 66 indicators across 7 domains.

The Warnecke model, a multilevel framework that highlights how institutional factors affect individual-level cancer risk (17), was used to reduce the list of 66 indicators down to those representing social context, social relationships, and physical context. To accomplish this, CSA characteristics explicitly identified in both the model and Vital Signs report were noted. This overlap included indicators such as racial/ethnic integration, employment, and social/economic gradient. After selecting the characteristics that were found in the framework, the remaining characteristics in the Vital Signs report were assessed for potential proxies of other factors referenced in the Warnecke Model. These proxies were validated with the scientific literature. This resulted in the inclusion of tree coverage, which has been shown to be a marker of social cohesion, as well as vacant housing, which has an inverse relationship with neighborhood stability (18, 19). Within-domain correlation between the indicators was also assessed.

### Statistical analyses

A series of descriptive statistics were conducted using STATA 12.1 (20). Individual-level characteristics provided by the MCR were evaluated, such as cancer type, mean age at diagnosis, tumor grade, and race.

Choropleth maps were created to shade CSAs for each cancer type by quintiles of cancer incidence. Spatial clusters (“hot spots”) were identified using the Getis-Ord Gi^*^ statistic and local Moran's I. By evaluating the local sum of cancer incidence in a CSA and its neighbors relative to the total sum in all of Baltimore City, this local spatial method measured pockets of spatial association that may have otherwise been obscured by global statistics (21).

A global ordinary least squares (OLS) regression model was then conducted for each cancer type to identify potential independent neighborhood-level variables that explained the geographic distribution of these clusters. The OLS model produces a linear fit that yields the smallest residuals and provides a single regression fit that explains which neighborhood characteristics potentially drive cancer to occur where it does. All of the described spatial analyses was conducted using ArcGIS 10.3 software (22).

The Johns Hopkins Bloomberg School of Public Health's Institutional Review Board and the Maryland Department of Health and Mental Hygiene's Institutional Review Board determined this study to be exempt research.

## Results

### Neighborhood characteristics

Of the original 66 indicators available through the Vital Signs report, the list was reduced to 16 indicators (Table 1) that best described social relationships as well as social and physical contexts of environments as defined in the Warnecke model. Since the selected indicators remained relatively stable within CSAs over the 2000–2010 study period as observed through visual inspection of overlapping margin of errors, the average across all available years for each CSA was used. Of the neighborhood-level covariates provided by the Vital Signs report, the highest correlations within a domain across the 16 indicators were seen between single female-headed households and percent African-American residents (0.79) as well as between single female-headed households and percent of households with < $25,000 income (0.80).

**Table 1:** Definitions of community statistical area characteristics.

**Indicator**	**Definition**
Females 50–74	Total number of female residents age 50–74 years
African-American	Percent of residents that identify themselves as being racially Black or African American (and ethnically non-Hispanic)
Racial Diversity Index	Percent chance that two people picked at random within an area will be of a different race/ethnicity. The higher the value, the more racially and ethnically diverse an area
Female-headed	Percent of female-headed households with own children aged 18 years and younger
< $25K	Percent of households earning < $25,000
Vacant	Percent of residential properties that have been classified as being vacant and abandoned
Housing violations	Percent of residential properties with housing violations (excluding vacants)
Crime	Total number of Part 1 crime incidents per 1,000 residents
Domestic violence	Total number of calls to emergency 911 for domestic violence per 1,000 residents
Teen birth	Total female teens aged 15–19 years that gave birth per 1,000 females aged 15–19 years
Employed	Percent of persons aged 16–64 years formally employed or self-employed
Businesses	Total number of businesses (both for-profit and non-profit)
Voted	Percent of persons who voted in the last general election
Dirty streets	Total number of service requests for dirty streets and alleys per 1,000 residents
Tree coverage	Percent of total land area comprised of tree canopy
Neighborhood associations	Total number of neighborhood associations and block clubs

### Cancer incidence and population characteristics

The study population consisted of 4,966 total cases across the three cancer sites of interest: breast (*n* = 3,466), cervical (*n* = 380), and colorectal (*n* = 1,120) cancer among 4,928 unique cases. There were 181 cases excluded from the analysis since their address could not be geocoded to Baltimore City. For most of these exclusions, the primary reasons consisted of having no street address available or residing in a county outside of Baltimore City.

The mean age at cancer diagnosis in the study population was 56.3 (*SD* = 11.2) years. However, the mean age at diagnosis significantly differed across cancer sites. Cervical cancer cases were diagnosed, on average, at a younger age of 50.0 (*SD* = 12.7) years. Colorectal cancer cases were the oldest of the three sites at 59.9 (*SD* = 10.1) years. Significant differences by cancer type were observed for cancer stage while the breakdown of White non-Hispanic vs. Black non-Hispanic cases was comparable across cancer types (data not presented).

**Figure 1:**
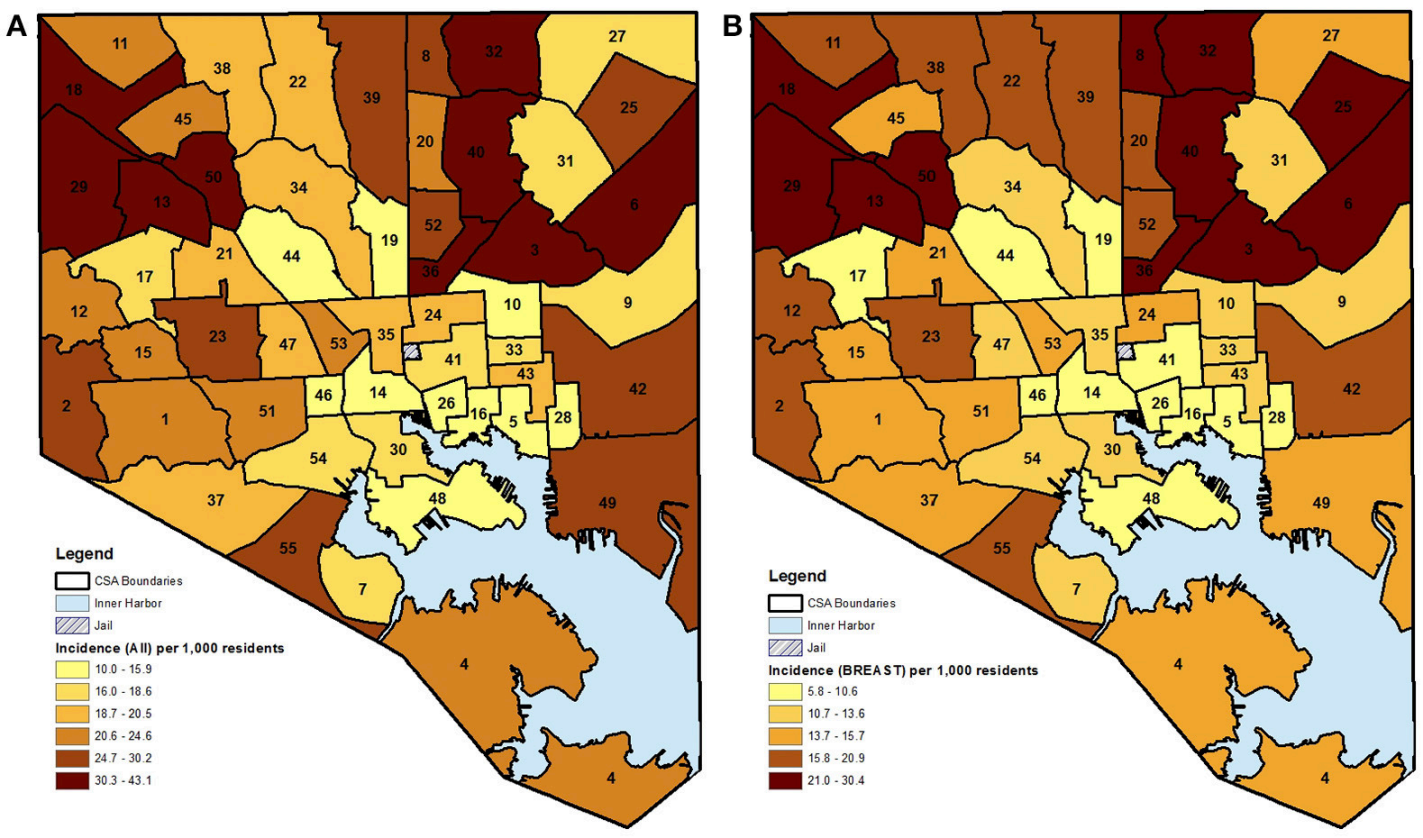
**(A,B)** CSA distribution shaded by quintile of female cancer incidence (all and breast) in Baltimore City, MD per 1,000 female residents aged 21–74 years, 2000–2010. (See Supplemental Table 1 for key to CSA ID numbers).

**Figure 2:**
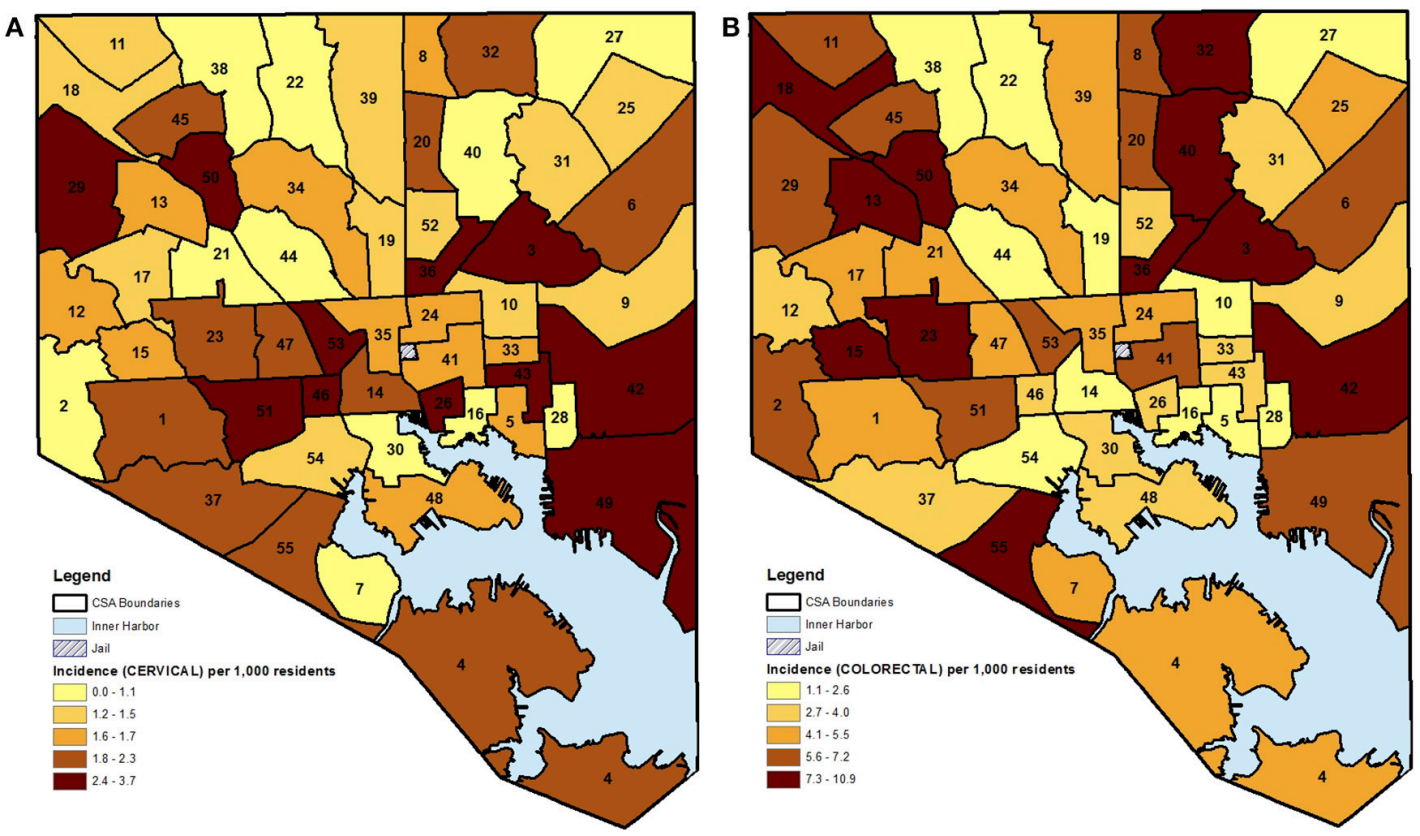
**(A,B)** CSA distribution shaded by quintile of female cancer incidence (cervical and colorectal) in Baltimore City, MD per 1,000 female residents aged 21–74 years, 2000–2010. (See Supplemental Table 1 for key to CSA ID numbers).

The choropleth maps demonstrated that the local spatial distribution of cancer incidence per 1,000 female residents aged 21–74 years varied greatly overall and by cancer type (Figures 1, 2). This spatial distribution remained mostly unchanged when restricting to female residents aged 50–74 years (data not presented). The number of female cases ranged from 27 to 191 diagnosed residents in any single geographic unit over the 10 year period. The CSA of Cedonia/Frankford yielded the most cancer cases while Dickeyville/Franklintown had the fewest. Breast cancer incidence appeared to aggregate in the northeast and northwest areas of Baltimore City while cervical cancer occurred in the southeast and southwest CSAs. The highest quintiles of colorectal cancer incidence were located in similar areas as breast cancer with additional high incidence in the southwestern CSA of Westport/Mount Winnans/Lakeland.

Table 2 visually summarizes the quintile data as a heatmap. The table clearly demonstrates that a CSA falling in the highest quintile for incidence in one cancer site did not necessarily fall in the highest quintile for another cancer site. For example, the Harbor East/Little Italy CSA was in the top 80th percentile for cervical cancer incidence while falling to the bottom 20% for breast cancer. Of the 55 CSAs, only 10 neighborhoods had each of the three cancer sites appear in the same quintile. Three of these CSAs, Belair-Edson, Midway/Coldstream, and Southern Park Heights, had all cancer incidence rates in the highest quintile. This result strengthens the argument that local drivers may go unnoticed if there is too much aggregation, such as collapsing across various primary tumor types, with the assumption that the same neighborhoods will be appear as high-risk for all cancer types.

**Table 2:** Heatmap summarizing the distribution of female cancer incidence (quintiles) and hot/cold spatial clusters by CSA for Baltimore City, 2000–2010.

		**Quintile distribution**			**Cluster spot**		
**ID**	**CSA**	**Breast**	**Cervical**	**Colorectal**	**Breast**	**Cervical**	**Colorectal**
1	Allendale/Irvington/S. Hilton						
2	Beechfield/Ten Hills/West						
3	Belair-Edison						
4	Brooklyn/Curtis Bay						
5	Canton						
6	Cedonia/Frankford						
7	Cherry Hill						
8	Chinquapin Park/Belvedere						
9	Claremont/Armistead						
10	Clifton-Berea						
11	Cross-Country/Cheswolde						
12	Dickeyville/Franklintown						
13	Dorchester/Ashburton						
14	Downtown/Seton Hill						
15	Edmonson Village						
16	Fells Point						
17	Forest Park/Walbrook						
18	Glen-Fallstaff						
19	Greater Charles Village/Barclay						
20	Greater Govans						
21	Greater Mondawmin						
22	Greater Roland Park/Poplar Hill						
23	Greater Rosemont						
24	Greenmount East						
25	Hamilton						
26	Harbor East/Little Italy						
27	Harford/Echodale						
28	Highlandtown						
29	Howard Park/West Arlington						
30	Inner Harbor/Federal Hill						
31	Lauraville						
32	Loch Raven						
33	Madison/East End						
34	Medfield/Hampden/Woodberry/Remington						
35	Midtown						
36	Midway/Coldstream						
37	Morrell Parkk/Violetville						
38	Mount Washington/Coldspring						
39	North Baltimore/Guilford/Homeland						
40	Northwood						
41	Oldtown/Middle East						
42	Orangeville/E. Highlandtown						
43	Patterson Park North & East						
44	Penn North/Reservoir Hill						
45	Pimlico/Arlington/Hilltop						
46	Poppleton/The Terraces/Hollins Market						
47	Sandtown-Winchester/Harlem Park						
48	South Baltimore						
49	Southeastern						
50	Southern Park Heights						
51	Southwest Baltimore						
52	The Waverlies						
53	Upton/Druid Heights						
54	Washington Village/Pigtown						
55	Westport/Mount Winans/Lakeland						
	**Lowest quintile**				**Highest quintile**		
**Quintile Key**						
	**Cold Spot-99% CI**			**Not Significant**			**Hot Spot-99% CI**
**Cluster Key**							

### Spatial analysis of cancer incidence

The results of the “hot spot” analysis assessed the statistical significance of the patterns observed in the choropleth maps. When comparing across the three cancer types, there were several differences in terms of where the significant hot and cold spots were located within Baltimore City (maps not shown, but data are presented in Table 2). The northern area of Baltimore City had a significant aggregation of breast cancer that was not seen in either cervical or colorectal cancer. In fact, the two “cold spots” yielded by the cervical cancer analysis were located in the area of high breast cancer incidence. Conversely, cervical cancer had a significant clustering of cases in the Canton CSA, which was a neighborhood with significantly lower breast and colorectal cancer incidence.

In order to explain the geographic location of “hot” and “cold spots” for each cancer type and to evaluate the potential drivers of the “hot spots,” such as age of the underlying population vs. other indicators, an OLS regression model was carried out using the 16 neighborhood-level characteristics of interest (Tables 3A,B). For all cancers, an increased proportion of African-Americans residents within a CSA as well as a decrease in number of businesses were associated with higher cancer incidence. When stratifying by cancer type, the significant associations with each of the CSA characteristics varied. Analysis indicated that every one percent increase in African-American residents resulted in breast cancer incidence increasing by 0.059 times per 1,000 female residents. It should be noted that given the use of the community-level variables in this analysis, cross-level inferences should not be made in order to avoid ecologic fallacy. Although there is a significant association between an increased proportion of African-American residents and increased breast cancer incidence, this does not necessarily mean that the African-American residents are the ones developing breast cancer.

**Table 3A:** Unadjusted ordinary least squares regression models for female cancer incidence by cancer site and neighborhood-level covariates, Baltimore City, 2000–2010.

**INDICATOR**	**All cancers**		**Breast**		**Cervical**		**Colorectal**	
	**Coefficient**	***p*-value**	**Coefficient**	***p*-value**	**Coefficient**	***p*-value**	**Coefficient**	***p*-value**
Females 50–74	−0.0001	0.945	0.0001	0.918	−0.0001	0.463	0.0003	0.604
African-American	0.100	0.004^*^	0.059	0.017^*^	0.006	0.126	0.031	0.001^*^
Racial Diversity Index	−0.078	0.566	−0.052	0.201	−0.002	0.787	−0.031	0.049^*^
Female-headed	−0.014	0.552	−0.059	0.377	0.024	0.009^*^	0.020	0.441
< $25K	0.036	0.552	−0.009	0.838	0.017	0.006^*^	0.028	0.107
Vacant	−0.140	0.229	−0.159	0.060	0.018	0.142	0.001	0.976
Housing violations	−0.326	0.577	−0.531	0.214	0.121	0.046^*^	0.084	0.622
Crime	−0.043	0.009^*^	−0.033	0.006^*^	0.001	0.642	−0.011	0.023^*^
Domestic violence	0.014	0.853	−0.032	0.561	0.026	< 0.004^*^	0.020	0.363
Teen birth	0.013	0.716	−0.018	0.482	0.013	< 0.001^*^	0.018	0.078
Employed	−0.024	0.816	0.050	0.506	−0.032	0.002^*^	−0.041	0.157
Businesses	−0.005	0.032^*^	−0.003	0.033^*^	0.000	0.999	−0.001	0.050
Voted	0.224	0.075	0.258	0.004^*^	0.224	0.075	0.004	0.915
Dirty streets	−0.016	0.417	−0.017	0.231	0.004	0.085	0.002	0.692
Tree coverage	0.110	0.100	0.121	0.013^*^	−0.017	0.013^*^	0.007	0.718
Neighborhood associations	0.065	0.760	−0.033	0.835	0.024	0.291	0.074	0.227

* *Statistically significant at p-value < 0.05 threshold*.

Notably, crime had an inverse relationship with cancer incidence indicating that CSAs with a higher cancer burden experienced less crime. One possible explanation is that high-crime areas tend to have younger populations. When evaluated within the context of Baltimore City CSAs, the neighborhood-level data did show a slight increase in crime rates as the proportion of residents between the ages of 25–64 years old also increased. As a result, cancer incidence would appear to be lower given that older individuals make up a smaller portion of the population. This significant association was observed in both breast and colorectal cancer. This relationship may not have been observed for cervical cancer due to the younger average age at diagnosis as compared to the other sites. Surprisingly, a higher proportion of voters, which is often a proxy for the sustainability and social capital of a neighborhood, appeared to be significantly associated with a greater breast cancer burden. This can likely be explained through voter participation occurring in more urbanized settings where voting facilities are more easily accessible, or this may also be due to the fact that older populations are more likely to vote. Overall, many of the significant associations observed in the unadjusted models were expected given the potential pathways these intermediate factors could affect cancer incidence. For cervical cancer, neighborhood characteristics, such as a high teen birth rate, reflected the behavioral risk factors of unprotected sex, which is a predictor of cervical cancer (Table 3A).

Each of these models was evaluated through several OLS diagnostic tools to determine fit as well as to avoid bias. None of the unadjusted spatial models were statistically significant for the Jarque-Bera statistic, which would have indicated that the residuals were not normally distributed. The significant covariates for each cancer site were integrated into their respective adjusted models (Table 3B). Also, while not significant in the unadjusted model, the number of females 50–74 years that lived within a CSA was included *a priori* in the adjusted model due to the known association between age and cancer risk. Most of the neighborhood covariates retained their statistical significance in the fully adjusted model, except for cervical cancer. The integration of the covariates in that case resulted in null associations. The final models were evaluated through the *R*-squared statistic, which demonstrated the proportion of the variability in the outcome that was explained by the model. Aggregating across all cancer types, the model fitting percent of females 50–74 years, percent of African-American residents, crime rate, and number of businesses, explained over one-third of the geographic distribution observed in the “hot spot” maps (*R*^2^ = 0.341).

**Table 3B:** Adjusted ordinary least squares regression models for female cancer incidence by cancer site and neighborhood-level covariates, Baltimore City, 2000–2010.

**Cancer type**	**Indicator**	**Coefficient**	***p*-value**	***R*-squared**
All cancers	Females 50–74 years	−0.005	0.012^*^	0.341
	% AA	0.144	< 0.001^*^
	Crime	−0.169	< 0.001^*^
	Businesses	0.017	0.002^*^
Breast cancer	Females 50–74 years	−0.003	0.058	0.349
	% AA	0.102	< 0.001^*^
	Crime	−0.100	0.005^*^
	Businesses	0.011	0.011^*^
	Voted	0.222	0.029^*^
	Tree coverage	−0.014	0.792
Cervical cancer	Females 50–74 years	0.0001	0.748	0.108
	Female headed	−0.011	0.470
	Housing violations	−0.036	0.728
	Domestic violence	0.023	0.278
	Teen births	−0.0001	0.982
	Employed	−0.012	0.600
	Tree coverage	0.006	0.502
	Neighborhood associations	0.032	0.279
Colorectal cancer	Females 50–74 years	−0.0001	0.894	0.108
	% AA	0.022	0.025^*^
	Crime	−0.008	0.084

* *Statistically significant at p-value < 0.05 threshold*.

## Discussion

Overall, the three primary findings were: (1) the existence of local variation in cancer incidence within Baltimore City female residents; (2) the noticeable differences in observed geographic distributions among breast, cervical, and colorectal cancer; and (3) the differences in statistically significant neighborhood characteristics that explained some geographic variation for each cancer type. A notable finding was the observed differences in the locations of spatial clusters when comparing across cancer types. Each primary cancer was anticipated to have distinct nuances given underlying differences. For example, cervical cancer cases are often diagnosed at earlier ages as compared to colorectal and breast cancer cases which might be impacted by the age distribution of the neighborhood's residents. The population structure of the neighborhoods in conjunction with other community-level characteristics likely resulted in some neighborhoods being identified as a “cold spot” for one cancer but a “hot spot” for another.

From these findings and the thematic maps generated for cancer incidence, there is an early indication that geography must be factored in when assessing disparities as well as deciding on the allocation of resources. However, even when neighborhood characteristics are ultimately incorporated into analyses, the results of this study demonstrate that the granularity of those area-level characteristics need to be carefully considered. When small area estimates are not taken into account, such as by aggregating across several counties for state-level rates, public health professionals run the risk of applying approaches that are costly and ineffective because they were implemented in the wrong neighborhood. Additionally, these findings solidify the notion that neighborhood factors are associated with different cancer types to different degrees, which may result in the solutions varying from one disease to the next.

These analyses have several strengths and limitations. One limitation is that neighborhood context is limited only to the residential environment, as defined by the address at the time of the cancer diagnosis. The concept of social determinants is an all-encompassing one that evaluates how health “is impacted by where and how we live, learn, work, and play” (23). This study fails to capture the additional resources or disadvantages an individual may be exposed to through the areas in which they work or socialize. For example, access to care or availability of cancer screening services within a neighborhood may impact the observed disease burden. This attribute was not available in the information collected by the Baltimore Neighborhood Indicators Alliance and thus could not be assessed in the analysis. Another limitation related to the residential address data is that the MCR did not have information on how long female cancer cases had resided at their reported address prior to their diagnosis. Since cancer is recognized as having a long latency period, an incident cancer case that only recently moved to her current residence in Baltimore City may not have been exposed long enough to her neighborhood to be a true representation of that area's association with cancer outcomes. However, in Baltimore City, over 80% of female residents between the ages of 21 and 74 years reported having the same residence the year before, which remained relatively stable from 2000 to 2010 (24). Most of the mobility within Baltimore City came from the younger age groups as this figure ranged from 65 to 78% among women between the ages of 21–33 years, which would have more of an impact on cervical cancer cases given it is diagnosed at an earlier age than colorectal and breast cancer cases.

Another aspect of this study that might be viewed as a limitation is the absence of individual-level characteristics, such as age and race, as well as health behaviors commonly associated with the development of cancer, such as smoking, in the OLS analyses. Without these variables, the models are limited in determining how much of the association seen between neighborhood characteristics and cancer incidence is due to individual-level factors vs. the independent effect of the CSA. It should be noted that given the use of the community-level variables in this analysis, cross-level inferences should not be made in order to avoid ecologic fallacy. For example, although there is a significant association between an increased proportion of African-American residents and increased breast cancer incidence, this does not necessarily mean that the African-American residents are the ones developing breast cancer. This question was beyond the scope of this analysis. The focus was to validly assess whether the social and physical environments of Baltimore City neighborhoods were associated with cancer incidence. This information is particularly relevant for future interventions that want to allocate resources and services to areas that have a higher absolute public health burden. Also, the reliance on neighborhood-level data rather than individual-level data is a realistic scenario encountered by health departments as well as local organizations in the development and operationalization of programs to address community health. While there was a reliance on neighborhood-level data, the impact of health policies initiated during the study period were not considered. For example, the creation of the Cigarette Restitution Fund Program in 2000 by the Maryland General Assembly was a state-wide initiative that aimed to address cancer prevention, detection, and treatment. The assessment of health programs that may have been initiated in small local communities along with quantifying their presence within the geographic boundaries of specific CSAs were beyond the scope of this analysis.

Despite these limitations, this study has a number of strengths. Firstly, it improves upon prior characterizations of neighborhood context by integrating community-level measures that have been collected on Baltimore City neighborhoods for over a decade (16). Previous studies have relied mostly on U.S. Census Bureau data, which are not extensive measures of the social and physical environments of communities. While others may have attempted to account for neighborhood context in their analyses, few have utilized community covariates that have the same breadth and depth as those tracked in Baltimore City. The use of these additional neighborhood data painted a more complete picture of where Baltimore City residents live while also providing potential suggestions as to neighborhood characteristics that could be collected more routinely in other cities.

Additionally, through the use of data curated by the Baltimore Neighborhood Indicators Alliance, the study was able to utilize the CSA as its geographic unit of analysis. The ability to focus on neighborhoods within a specific county allowed for the calculation of small area estimates, which improved statistical precision while also maintaining geographic resolution. Small area estimates increase the likelihood of homogeneity across the neighborhood characteristics within the geographic boundaries and thus increase the validity of the associations. There have been other studies on geographic variation across broader regions that may have overlooked the local patterns of disease, especially in the absence of unique community data utilized in this study (25, 26). There is also cultural relevance of the geographic boundaries utilized. Residents are often unaware of the census tracts they live in, which is another small-scale administrative unit often used in geospatial analyses. Through the Baltimore Neighborhood Indicators Alliance, Baltimore City residents have grown accustomed to hearing about the quality of life within CSA boundaries due to the measures that have been collected over the years. As a result, CSAs might be easier to identify with and subsequently intervene on since there is a better grasp of the population's composition and context.

This study took a transdisciplinary approach to broadening the medical model of cancer research. By taking into account neighborhood residence, the findings help shed light on the complexity of cancer incidence and cancer disparities. This provides additional insight as to the research areas that could join in collaborative efforts to address inequities in health outcomes among subgroups. Based on the results, there is compelling evidence to pursue further research on the association of neighborhood factors with the geographic distribution of cancer incidence beyond just Baltimore City. The findings make the case that there is an opportunity to create effective geographically tailored cancer services, which has been accomplished in other communities (27–29).

More specifically, the study findings suggest that developing geographically tailored interventions to address cancer should target specific cancer types. There may have likely been past cancer-related interventions that, although might have appeared ineffective, were actually misallocated to populations and geographic locations that did not fit the targeted risk profile. Upon observing the different associations with neighborhood characteristics across cancer types, it would be prudent to take careful consideration when attempting to intervene through more upstream avenues. Previously, the assumption might have been made that addressing a particular social determinant or improving a specific contextual neighborhood characteristic would result in downstream improvements for all health outcomes. The unique community measures in this study provide a new perspective on the relationship between social determinants at the neighborhood level and cancer incidence. These analyses provide early evidence that neighborhood factors might affect exposure-outcome associations in different ways.

Overall, the study demonstrated that area differences might go unnoticed when leveraging larger geographic boundaries, such as county—or state-level borders. This is even more likely to occur when those areas are comprised of a widely heterogeneous population with varying levels of risk factors. Future research should begin expanding the types of neighborhood metrics collected and used in the study of geographic cancer disparities. Additionally, it should be evaluated whether the distribution and proximity of resources align with the location of disease burden. If it does not, spatial statistics can assist in guiding how those prevention and treatment programs are redirected.

## Author contributions

All authors listed have made a substantial, direct and intellectual contribution to the work, and approved it for publication.

### Conflict of interest statement

The authors declare that the research was conducted in the absence of any commercial or financial relationships that could be construed as a potential conflict of interest.
