# Supplementary material for: Evaluating Neighborhood Correlates and Geospatial Distribution of Breast, Cervical, and Colorectal Cancer Incidence
Source: Front Oncol. 2018 Oct 30;8:471. doi: 10.3389/fonc.2018.00471 (PMC6218580; doi:10.3389/fonc.2018.00471)
Supplement: Supplementary file 1 [file Table_1.pdf]

Supplemental Table 1: Key for Community Statistical Area map

Allendale/Irvington/S. Hilton	1	Howard Park/West Arlington	29
Beechfield/Ten Hills/West	2	Inner Harbor/Federal Hill	30
Belair-Edison	3	Lauraville	31
Brooklyn/Curtis Bay	4	Loch Raven	32
Canton	5	Madison/East End	33
Cedonia/Frankford	6	Medfield/Hampden/Woodberry/Remington	34
Cherry Hill	7	Midtown	35
Chinquapin Park/Belvedere	8	Midway/Coldstream	36
Claremont/Armistead	9	Morrell Parkk/Violetville	37
Clifton-Berea	10	Mount Washington/Coldspring	38
Cross-Country/Cheswolde	11	North Baltimore/Guilford/Homeland	39
Dickeyville/Franklintown	12	Northwood	40
Dorchester/Ashburton	13	Oldtown/Middle East	41
Downtown/Seton Hill	14	Orangeville/E. Highlandtown	42
Edmonson Village	15	Patterson Park North & East	43
Fells Point	16	Penn North/Reservoir Hill	44
Forest Park/Walbrook	17	Pimlico/Arlington/Hilltop	45
Glen-Fallstaff	18	Poppleton/The Terraces/Hollins Market	46
Greater Charles Village/Barclay	19	Sandtown-Winchester/Harlem Park	47
Greater Govans	20	South Baltimore	48
Greater Mondawmin	21	Southeastern	49
Greater Roland Park/Poplar Hill	22	Southern Park Heights	50
Greater Rosemont	23	Southwest Baltimore	51
Greenmount East	24	The Waverlies	52
Hamilton	25	Upton/Druid Heights	53
Harbor East/Little Italy	26	Washington Village/Pigtown	54
Harford/Echodale	27	Westport/Mount Winans/Lakeland	55
Highlandtown	28		
